# Optimal and continuous anaemia control in a cohort of dialysis patients in Switzerland

**DOI:** 10.1186/1471-2369-9-16

**Published:** 2008-12-11

**Authors:** Claudine M Mathieu, Daniel Teta, Nathalie Lötscher, Dela Golshayan, Luca Gabutti, Denes Kiss, Pierre-Yves Martin, Michel Burnier

**Affiliations:** 1 University Hospital CHUV, Department of Nephrology, Lausanne, Switzerland; 2 Roche (Pharma) Switzerland Ltd, Reinach, Switzerland; 3 Ospedale Regionale, Department of Nephrology, Locarno, Switzerland; 4 Kantonsspital, Department of Nephrology, Liestal, Switzerland; 5 University Hospital HCUG, Department of Nephrology, Geneva, Switzerland

## Abstract

**Background:**

Guidelines for the management of anaemia in patients with chronic kidney disease (CKD) recommend a minimal haemoglobin (Hb) target of 11 g/dL. Recent surveys indicate that this requirement is not met in many patients in Europe. In most studies, Hb is only assessed over a short-term period. The aim of this study was to examine the control of anaemia over a continuous long-term period in Switzerland.

**Methods:**

A prospective multi-centre observational study was conducted in dialysed patients treated with recombinant human epoetin (EPO) beta, over a one-year follow-up period, with monthly assessments of anaemia parameters.

**Results:**

Three hundred and fifty patients from 27 centres, representing 14% of the dialysis population in Switzerland, were included. Mean Hb was 11.9 ± 1.0 g/dL, and remained stable over time. Eighty-five % of the patients achieved mean Hb ≥ 11 g/dL. Mean EPO dose was 155 ± 118 IU/kg/week, being delivered mostly by subcutaneous route (64–71%). Mean serum ferritin and transferrin saturation were 435 ± 253 μg/L and 30 ± 11%, respectively. At month 12, adequate iron stores were found in 72.5% of patients, whereas absolute and functional iron deficiencies were observed in only 5.1% and 17.8%, respectively. Multivariate analysis showed that diabetes unexpectedly influenced Hb towards higher levels (12.1 ± 0.9 g/dL; p = 0.02). One year survival was significantly higher in patients with Hb ≥ 11 g/dL than in those with Hb <11 g/dL (19.7% vs 7.3%, p = 0.006).

**Conclusion:**

In comparison to European studies of reference, this survey shows a remarkable and continuous control of anaemia in Swiss dialysis centres. These results were reached through moderately high EPO doses, mostly given subcutaneously, and careful iron therapy management.

## Background

Effective anaemia control in dialysis patients is associated with many benefits, including lower mortality, morbidity and better quality of life [1-6]. Despite significant improvement in the last few years, recent surveys, such as the European Survey in Anaemia Management (ESAM) 2003 [7] and the Dialysis Outcomes and Practice Pattern Study II (DOPPS II) [8], show that the minimal haemoglobin (Hb) target of 11 g/dL, proposed by the European Best Practice Guidelines (EBPG) [9] and the Kidney Disease Outcomes Quality Initiative (K/DOQI) [10], was not achieved in many patients. In Switzerland, only few data on the control of anaemia in dialysed patients is available. In ESAM 1998 [11], Switzerland achieved mean Hb of 11.7 g/dL (mean Hb of all countries was 11.4 g/dL). In ESAM 1998 and 2003, the proportion of patients above the minimal target of Hb >11 g/dL in this country were 65.1 and 78.9%, respectively, leaving many patients under the target. It is noteworthy that ESAM 2003 was based on a 1 day assessment, whereas data from ESAM 1998 has been collected during a six-months follow-up period. It is known that Hb is fluctuating during long term observations in dialysed patients [12-14]. Thus, we feel it would be meaningful to study: A) the quality of anaemia control in dialysed patients in Switzerland; B) if the control of anaemia and its treatment parameters could be maintained over a long period of time, C) if co-morbidities may modulate Hb level and EPO dose, and D) if anaemia management has improved when compared with earlier assessments.

## Methods

### Study design and data collection

**Figure 1:**
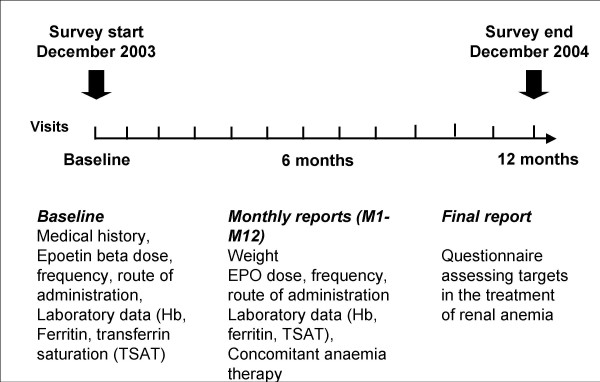
**Study design**. This was a prospective, observational survey in selected patients lasting from December 2003 (for the first patients included) to March 2005 (for the last patients included). Each single patient was observed during 12 months. Demographic and medical parameters, as specified in the figure, were collected at baseline, and then on a monthly basis.

This study was designed as a prospective, non-randomized, observational survey. Patients were recruited from November 2002 to March 2004. For each patient, the observation period lasted 12 months. A concise, four-pages report form was used for collecting baseline demographic data, clinical and laboratory parameters, as well as anaemia treatment modalities at study entry, and then monthly. The follow-up was only interrupted in the event of a death, a renal transplantation, or if the patient was lost to follow-up. Details of the study design and data collection are summarized in figure 1. The recruitment was performed in 27 dialysis centres, on a voluntary basis. There were 3 university-based centres, 17 regional-based and 7 private dialysis centres. According to Swiss law, an informed consent is not needed for this type of survey. No change in therapeutic strategy was requested during the observational period. Additionally, a questionnaire was sent to the participating centres, asking for centre-specific targets for anaemia treatment such as Hb, serum ferritin and transferrin saturation (TSAT).

Three categories of iron status were defined: 1) absolute iron deficiency (ferritin <100 μg/L); 2) functional iron deficiency (ferritin ≥ 100 μg/L and TSAT <20%); and 3) adequate iron status (ferritin ≥ 100 < 800 μg/L and TSAT ≥ 20%).

### Selection of patients

The study population were adult patients undergoing maintenance renal replacement therapy, by either haemodialysis, haemodiafiltration or peritoneal dialysis. No minimal time on dialysis was requested. Adequate iron status (ferritin ≥ 200 μg/l and TSAT ≥ 20%) was recommended but not mandatory to be enrolled for the study. Inclusion criteria were: i) age of ≥ 18 years; ii) either epoetin (EPO) pre-treated (any type of EPO) or EPO-naive patients with newly diagnosed renal anaemia; iii) patients had to be exclusively on EPO beta during the course of the study. Exclusion criteria were the following: unstable angina pectoris, untreated uncontrolled hypertension, haemoglobinopathy, haemolysis, gastrointestinal bleeding, acute infection or unstable systemic inflammatory disease, epilepsy, pregnancy, lactation, deficiency of vitamin B12 (<200 ng/L), deficiency of acid folic (<2 μg/L), planned surgery during the survey period (except fistula surgery), known hypersensitivity to EPO beta.

Participating centres were asked to include as many patients as possible, on a voluntary basis, meeting the inclusion/exclusion criteria. Ten dialysis centres included more than 80% of all their patients treated with epoetin beta, and 15 centres in total included more than 60% of their patients receiving epoetin beta, contributing to 71% of the patients included in this survey.

### Data analysis

Simple statistical analyses were performed in Excel 2002 and for more complex statistics the SAS statistical program version 8.2 (SAS, Institute, Cary NC, USA) was used. Standard descriptive statistics (mean, standard deviation, confidence interval) were calculated for all variables including Hb, EPO dose, serum ferritin and TSAT. Means were calculated on an individual basis (per patient) for each month and thereafter and thereafter the means of the monthly data were used to calculate the one year average mean value for example for hemoglobin. Categorical variables were presented in absolute number and percentage and were compared with the X^2^-test whereas continuous variables with the Student's t-test or Wilcoxon two-sample test, where appropriate. The ANOVA model was used for the efficacy comparison of the different routes of administration. The Kaplan Meier analysis was taken to calculate survival probabilities. In the patients whose follow-up was interrupted, the data were collected and analysed until the last observation. The reasons for the interruption of follow-up were: death (33), transplantation [7], unknown reason [1]. Rare intermediate missing values of patients (Hb, body weight, EPO dose, administration frequency, route of administration), who received at least one survey medication, were replaced by using the last observation carried forward method (LOCF).

Univariate analysis was performed for Hb and EPO dose regarding concomitant diseases, diagnosis, age and gender. A multivariate analysis was performed which included the covariates with p < 0.05 in the univariate analysis. All statistical tests were two-sided and the significance was proofed on a 0.05 p-level.

## Results

### Demographics and baseline characteristics

Three hundred and seventy patients from 27 dialysis centres were initially recruited. Twenty patients were excluded, because of major surgery (n = 4), bleeding (n = 2), and missing data (n = 14). Three hundred and fifty patients were finally analysed, representing 14% of the total dialysis population of Switzerland. Patients characteristics are shown in table 1. Causes of end stage renal disease (ESRD) and co-morbidities are described in table 2 and 3, respectively. Hypertension, diabetes and ischaemic heart disease were the most common concomitant pathologies; these findings are comparable to ESAM 2003, though in the latter, 20.2% of the patients suffered of peripheral vascular disease, compared to only 4.6% in our survey.

**Table 1:** Patients characteristics at baseline

**(n = 350 patients)**	**Mean ± SD**
Age (years)	63.9 ± 14.5
Range	18–91
Female (%)	145 (41.4)
Male (%)	205 (58.5)
Haemodialysis/Haemodiafiltration (%)	332 (94.9)
Peritoneal dialysis (%)	18 (5.1)
EPO pre-treated (%)	330 (94.3)
EPO naive (%)	20 (5.7)
Body weight (kg)	69.9 ± 15.3
Range	40–129
Haemoglobin (g/dL)	11.7 ± 1.4
Range	6.8–15.5
Serum ferritin (μg/L)	404 ± 293
	
TSAT (%)	31 ± 15

**Table 2:** Causes of end-stage renal disease

**Primary Renal Diagnosis**	**n (%)**
Glomerulonephritis	78 (22.3)
Diabetic nephropathy	75 (21.3)
Hypertension/vascular nephropathy	74 (21.1)
Pyelonephritis/interstitial nephritis	61 (17.4)
Polycystic kidney disease	27 (7.7)
Unclear/Multifactorial	13 (3.7)
Miscellaneous causes	11 (3.1)
Malignancies	9 (2.6)
Missing	12 (3.4)

Patients could be recorded with more than one primary diagnosis.

**Table 3:** Co-morbidities

	**n**	**%**
Hypertension	213	60.9
Diabetes^1^	96	27.4
Coronary artery disease	88	25.1
Congestive heart failure	55	15.7
Other cardiac diseases^2^	17	4.9
Vascular diseases^3^	16	4.6
Cancer (ca)^4^	14	4.0
Metabolic disorders^5^	7	2.0
Hepatological disorders^6^	7	2.0
Chronic obstructive pulmonary disease (COPD)	5	1.4
Hyperparathyroidism	4	1.1
Others (nephrectomy/ciclosporine nephropathy)	3	0.9
Amyloidosis	1	0.2

^1 ^Diabetes (overall): including reported as primary diagnosis (n = 75 patients)

^2 ^Aneurysms (2); Mitral insufficiency (3); Paroxysmal tachycardia (1); Pericarditis (1); Valvulopathy (1); Replacement of aortic valve (1) Ischemic cardiopathy (8)

^3 ^Arteriosclerosis, arteriopathy, polyarteriovascular disease (12); Cerebrovascular disorder (3); bleeding(1);

^4 ^Cancer: reported as concomitant diseases: prostate ca (2); ear-nose-throat ca (1); stomach ca (1); others and unknown (10)

^5^Obesity(3); Hypercholesterolaemia (1); Thyroiditis (1); Gout (2)

^6^Liver transplantation (1); Liver cirrhosis (4); Pancreatitis (2)

Patients could be recorded under more than one co-morbidity.

### Longitudinal assessment of Hb, iron status and EPO dose

**Figure 2:**
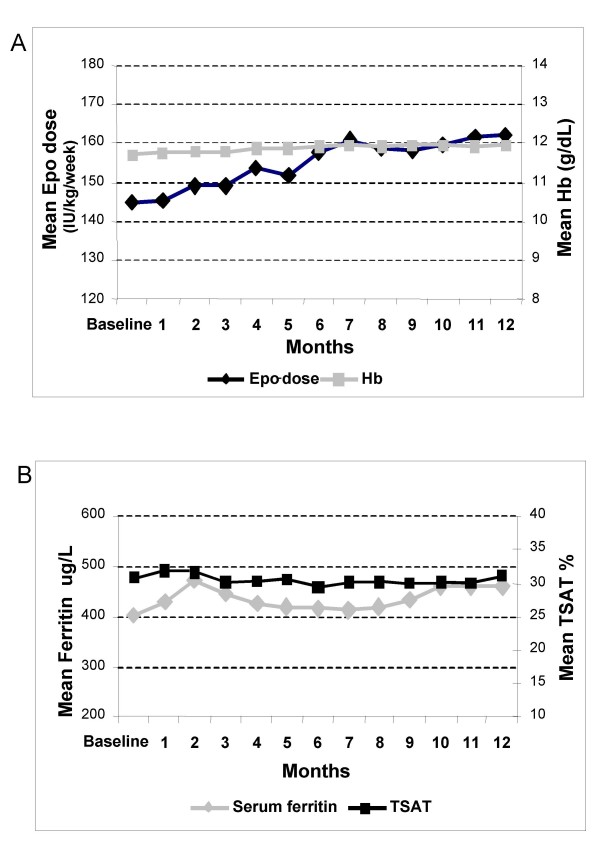
**Monthly variations**: month to month variations of mean Hb and EPO dose (upper panel A) and iron status (ferritin and transferrin saturation, TSAT, lower panel B) over the one-year observation period.

Month to month assessments of Hb, EPO dose and iron status, over the one-year observation period, are summarized graphically in figure 2. The overall mean Hb concentration based on the means of per patient values was 11.9 ± 1.0 g/dL, with a slight trend to increase over time, although not significantly. Mean weekly EPO dose was 155 ± 118 IU/kg/week, with a greater trend to increase from baseline to month 12 (but not significant). Mean serum ferritin and TSAT were 435 ± 253 μg/L and 30 ± 11%, respectively, with no significant variations.

**Figure 3:**
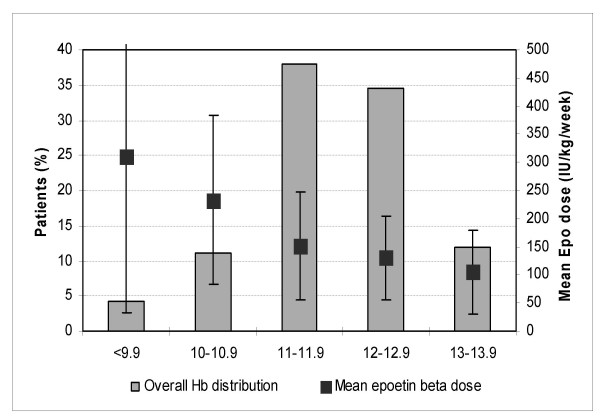
**Mean Hb distribution over the follow-up period**. Mean hemoglobin and EPO doses were first calculated on a per patient basis monthly and then over one year. Finally, the mean was calculated for the entire population with one mean per patient. The same was done for the EPO doses. Then, 5 Hb subgroups have been defined, according to sections of 1 g/dL.

The overall distribution of mean Hb is shown in figure 3. Eighty five % of the patients achieved mean Hb ≥ 11 g/dL and 44% had mean Hb ≥ 12 g/dL. The relationship between Hb and EPO dose was examined. The requirement of EPO for each section of mean Hb level is shown in the figure. Mean weekly EPO dose was significantly lower (p < 0.0001) in patients with mean Hb ≥ 11 g/dL (137 ± 86 IU/kg/week; 95% CI: 127–146 IU/kg/week) as compared to patients with mean Hb <11 g/dL (255 ± 194 IU/kg/week; 95% CI: 203–307 IU/kg/week). A significant negative correlation was found between overall mean EPO dose and mean Hb level (r = -0.21; p < 0.0001).

### Effect of gender, age and co-morbidities

Mean Hb (calculated as described above in figure 3) in males was significantly higher than in females (12.0 ± 0.1 versus 11.7 ± 1.0 g/dL, p = 0.01). Mean EPO dose was lower in men (146 ± 112 IU/kg/week) than in women (167 ± 124 IU/kg/week), but this difference was not significant (p = 0.11).

The influence of age was not significant. However, mean EPO dose tended to be higher in women younger than 50 years compared to women older than 50 (199 ± 153 versus 159 ± 116 IU/kg/week; p = 0.21).

**Figure 4:**
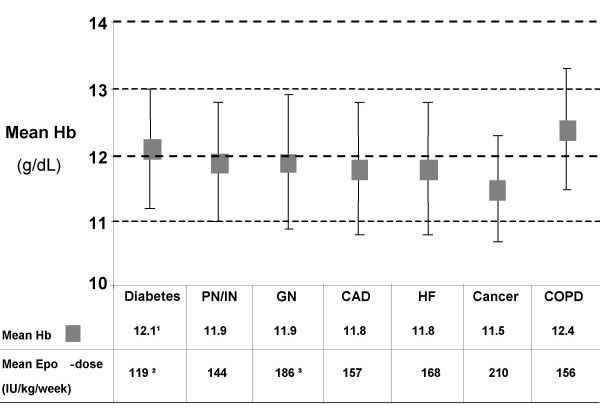
**Hb and EPO dose, according to co-morbidities and/or primary renal diagnosis**. The mean ± SD of Hb and EPO doses as calculated in figure 3 are shown according to comorbidities or the primary renal diagnosis. Diabetes: n = 96; pyelonephritis/interstitial nephritis (PN/IN): n = 61; glomerulonephritis (GN): n = 78; coronary artery disease (CAD): n = 88; congestive heart failure (HF): n = 55; cancer (ca): n = 14 (prostate ca (n = 2), ear-nose-throat ca (n = 1), stomach ca (n = 1), others ca non specified (n = 10); chronic obstructive pulmonary disease (COPD): n = 5. ^1^p = 0.004; ^2^p = 0.003; ^3^p = 0.008.

The influence of co-morbidities and primary renal diagnosis on mean Hb and EPO dose is shown in figure 4. Mean Hb was ≥ 11 g/dL in all subgroups, but varied according to co-morbidities and specific renal diagnosis. In multivariate analysis, diabetes was found to influence Hb towards a higher level (p = 0.004) and EPO requirements towards lower doses (p = 0.0004). Glomerulonephritis as a primary renal diagnosis influenced EPO towards a higher dose (p = 0.008), but not Hb.

### EPO administration and dose

At baseline, 71.1% of the patients received EPO beta subcutaneously. The proportion of intravenous administration tended to increase from 29 to 36% over time. A stable proportion of 68 to 70% of patients were treated with a once weekly regimen. Mean Hb was identical in the patients treated by intravenous or subcutaneous route. However, EPO dose was significantly lower in favour of the subcutaneous administration (114 ± 14 versus 187 ± 25 IU/kg/week, p = 0.035). For an identical mean Hb of 11.9 g/dL, patients treated once a week required a significantly lower EPO dose compared to patients treated 2 or 3 times per week (129 ± 103 versus 229 ± 105 IU/kg/week, p < 0.0001). This result is not surprising and only reflects the way epoetin beta is prescribed by Swiss nephrologists: low doses are generally given once a week, whereas higher doses (>10'000 IU/week) are divided into 2 or 3 injections.

Fifteen patients (4.3%) were found to be resistant to epoetin beta, defined by EPO doses higher than 300 IU/kg/week and Hb <11 g/dL. The co-morbidities of these patients were the following: coronary artery disease [4], heart failure [3], other cardiac diseases [1], diabetes[2], cancer [2], hypertension [4], liver disorder [1], chronic inflammation [1].

### Iron administration, iron status and adjuvant treatment of anaemia

In addition to EPO therapy, 97% of the patients received at baseline anti-anaemia adjuvant medication. Two hundred and thirty-two (68%) patients received iron substitution, intravenously (n = 187), orally (n = 21) or not specified (n = 24). About 13% of the patients received vitamins (including vitamin B12, vitamin C and folic acid). Transfusions were required in 8 (2.2%) patients.

Ninety-two, 91 and 92% of the patients had serum ferritin of ≥ 100 μg/L at baseline, month 6 and month 12, respectively. The percentage of patients with absolute iron deficiency ranged from 5.1 to 9.7% at any time during the observation period. At month 12, adequate iron status was found in 72.5% and functional iron deficiency in 17.8% of patients. The upper recommended limit of 800 μg/L was exceeded in 7.1% of the patients in this survey, ranging from 5.7 to 11.1% over the 12-months observation period. Sixty % of our patients were within the optimal ferritin target (200–500 μg/L) recommended by the EBPG.

**Figure 5:**
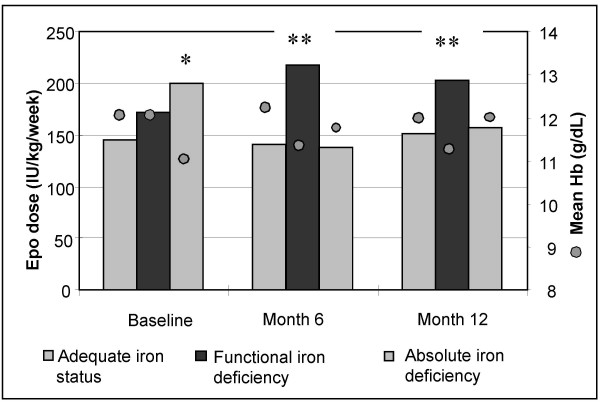
**Influence of iron status on Hb and EPO dose**. The iron status was divided into 3 categories, according to ferritin and TSAT (as defined in the text). Values at baseline, month 6 and 12 are shown. * Significant difference observed between patients with "adequate iron status" and "absolute iron deficiency" at baseline. ** Significant difference observed between patients with "functional iron deficiency" and "adequate iron stores" at month 6 (p = 0.0004 for Hb and p = 0.008 for the EPO dose) and month 12 (p = 0.03 for Hb, difference not significant for EPO dose).

Mean EPO dose and mean Hb level with respect to iron status are shown in figure 5. Overall, patients with "adequate iron status" had the highest Hb with the lowest EPO dose. At month 6 and 12, patients with "absolute iron deficiency" increased their Hb (p = 0.0004 at month 6, and 0.03 at month 12) compared to baseline values. These patients also needed less EPO (p = 0.008 at month 6 and p = ns at month 12, vs at baseline). The group "functional iron deficiency" had the lowest Hb level at month 6 and 12, despite the use of the highest EPO dose (>200 IU/kg/week).

### Analysis of mortality: effect of Hb and EPO dose

Thirty-three patients (9.4%) died during the follow-up. Patients with mean Hb ≥ 11 g/dL at baseline had a significantly better survival compared to patients with mean Hb < 11 g/dL (mortality rate: 7.3% vs 19.7%, respectively; p = 0.006). Patients with mean Hb ≥ 12 g/dL at baseline showed a mortality rate of 7% at one year, whereas those with mean Hb < 12 g/dL had a mortality rate of 11.5%, but this difference did not reach statistical significance.

As seen in figure 4, Hb was partially determined by co-morbidities, which represents a non modifiable factor for physicians. As Hb level was associated with mortality, we questioned whether EPO dose, a physician-dependent component of anaemia control, was associated with mortality, regardless of Hb. Patients were stratified into 4 categories: Patients with Hb ≥ 11 g/dL and EPO dose ≥ 200 IU/kg/week; patients with Hb ≥ 11 g/dL and EPO dose < 200 IU/kg/week; patients with Hb < 11 g/dL and EPO dose ≥ 200 IU/kg/week; and patients with Hb < 11 g/dL and EPO dose < 200 IU/kg/week. Survival rates were analysed according to these categories. Survival rate was in favour of patients with EPO doses lower than 200 IU/kg/week and Hb greater than 11 g/dL, but this observation failed to reach statistical significance (log rank test: p = 0.2). The same analysis was performed at a cut-off EPO dose of 300 IU/kg/week, showing a nearly significant lower survival probability in patients with lower Hb levels (<11 g/dL) and EPO dose >300 IU/kg/week (log-rank test: p = 0.064).

### Centre-specific target for Hb and iron status

In order to understand how this optimal anaemia control was achieved, nephrologists involved in the survey were asked about their personal anaemia management practice, by means of a questionnaire. For Hb, all centres aimed a target above 11 g/dL, with a range from 11 to 13.5 g/dL. In 15 of 27 centres (55.5%), target Hb was >12 g/dL. Regarding serum ferritin, 26 centres aimed values > 200 μg/L and 1 centre > 150 μg/L. However, 30% of the centres did not define upper limits as proposed by the EBGP. Fourteen centres (51.8%) aimed a target range from 200 to 500 μg/L as recommended by the EBPG. For TSAT, 18 centres indicated a target range from 20 to 40%.

## Discussion

With respect to ESAM 1998 [11], ESAM 2003 [7] and DOPPS II [8], this survey demonstrates an optimal anaemia control, obtained in 85% of patients in a cohort of dialysed patients in Switzerland followed prospectively for 12 months. Mean Hb of 11.9 g/dL was remarkably stable throughout the whole observation period.

ESAM 1998 [11] found an overall mean Hb value of 11.4 g/dL with only 49% of the patients reaching the target level. In ESAM 2003 [7], mean Hb levels were higher than in 1998 (mean Hb of 11.5 g/dL with 66.1% of patients having Hb>11 g/dL) in all but two countries. Recent data from the DOPPS II study [8] shows a mean Hb concentration ranging from 10.1 g/dL in Japan to 12.0 g/dL in Sweden, but with important percentages of patients not meeting the recommended target, even for countries with the highest mean Hb (Sweden: mean Hb 12.0 g/dL, 23% of the patients with Hb<11.g/dL; United States: mean Hb 11.7 g/dL, 27% of patients with Hb<11 g/dL; Spain: mean Hb 11.7 g/dL, 31% of patients with Hb<11 g/dL).

In both ESAM studies Switzerland showed a better Hb control compared to other countries. Looking at previous assessments in Switzerland, the current survey suggests an additional improvement from earlier studies regarding both Hb (mean Hb of 11.7 g/dL in ESAM 1998 and 11.9 g/dL in the current study) and the percentage of patients with Hb > 11 g/dL (65.1%, 78.9% and 85% in ESAM 1998, ESAM 2003 and the current study, respectively). It must be stated however that the differences in the nature and design in these studies require cautious interpretation regarding such an improvement. Nonetheless, the current results indicate that Swiss nephrologists have fully integrated EBGP recommendations published in 1999 [11]. The revised version of these guidelines (2004) [9] which proposes to tailor Hb from 11 to 14 g/dL according to co-morbidities, age and other factors, was released during the course of the present survey. It is unlikely to have influenced Hb management in this study. Another Swiss trial, prompted in 2005, is currently examining the impact of the revised EBPG on physician's practice.

Several factors appear to have contributed to these good results: first, our study shows a moderately high mean EPO beta dose, slightly above the upper limit of EBPG recommendations (50–150 IU/kg/week), with a trend to increase over the 12-months observation period. It is difficult to compare EPO doses between studies focussing on anaemia management, because in ESAM 2003 [7] and DOPPS II [8], EPO doses were not normalized to body weight. Nevertheless, with reference to the mean body weight of our patients, we calculated that mean EPO beta dose was 10'835 IU/week, which was higher than mean EPO used in ESAM 2003 (8'565 IU/week), but comparable to mean EPO doses from 12 countries in DOPPS II (ranging from 5'297 IU/week in Japan to 17'360 IU/week in the US). ESAM 2003 already showed increased mean EPO doses compared to ESAM 1998 [11] in all participating countries, certainly explaining most of the improvement of anaemia control.

Second, the majority (70%) of our patients received EPO subcutaneously compared to only 22.5% of the patients in ESAM 2003. Even though mean Hb was comparable in both ways of administration in our study, mean EPO dose was significantly lower for subcutaneous than intravenous administration. This finding confirms the better efficacy of the subcutaneous administration of EPO beta. DOPPS II [8] also reported an EPO dose reduction of about 14% with subcutaneous administration, though a difference of only 3% was reported in the US. ESAM 2003 showed that the differences in EPO doses between subcutaneous and intravenous administration were minimal for Hb values >11 g/dL. However, for the intravenous group, EPO doses were substantially higher for lower Hb values. It is thus conceivable that the major subcutaneous use of EPO in our study may has contributed to the current results.

Third, an adequate iron status is a crucial factor for achieving a satisfactory response to EPO therapy. Almost 73% of our patients had an adequate iron status. Only a small proportion of the patients (< 10%) had absolute iron deficiency and only 17.8% of patients had functional iron deficiency. Less than 10% had excessive iron stores. In the DOPPS study [8], a larger proportion of patients (35–40%) had TSAT < 20%, even though most of them were administered intravenous iron. In ESAM 2003, only 48% of the patients had adequate iron status and 31% had TSAT values below 20% [7]. In order to understand how nephrologists involved in our study managed iron deficiency, we analysed whether the patients with absolute and functional iron deficiency at baseline were corrected during the follow-up. During the whole observation, mean Hb level was lower and EPO dose higher in patients with "absolute iron deficiency" compared to the group with "adequate iron status". However, mean Hb of the group "absolute iron deficiency" increased from baseline to month 6 and 12, in parallel to a decrease of EPO requirements, suggesting that physicians attempted to correct for iron deficit. Thus, the management of iron appeared to be adequate. In contrast, we did not observe any Hb improvement over time in patients with" functional iron deficiency", despite increased use of EPO: this group had overall the lowest Hb (except at baseline, month 3, 4 and 8) together with the highest dose of EPO (except at baseline). As already shown in ESAM 1998 [11] and 2003 [7], functional iron deficiency has a stronger negative impact on Hb response rates than absolute iron deficiency. This observation justifies special attention on functional iron deficiency in the future, especially with respect to the role of adjuvant therapy such as vitamin C and carnitin. Finally, the questionnaire analysis shows that Swiss nephrologists generally refer to EBPG, even if the Hb target has been higher than recommended for about half of the physicians involved. This strategy may have driven some difficult patients towards a better control.

In order to identify factors potentially affecting anaemia management, we assessed the effect of age, gender, etiologies of end stage renal disease (ESRD) and co-morbidities on Hb level and EPO dose. Gender had a significant impact on Hb, but not on EPO dose. However, we observed a tendency for females to benefit from higher EPO doses, suggesting a fair attempt by nephrologists to equalize Hb between males and females. There was no obvious effect of age. However, younger age (<50 years) in women tended to influence Hb towards lower levels and, as a possible physician's response, towards higher EPO doses. In addition, older men (>60 years) tended to have lower Hb than younger men. In ESAM 2003 [7], women also had slightly lower mean Hb concentration than men, without differences in EPO dose after adjustment for Hb levels. Age had little effect on Hb and EPO dose, except for patients aged 70–79 years. In the latter group, Hb response rate and mean EPO dose were actually below average. In comparison, DOPPS II [8] showed significantly greater Hb for men, specially for the age group >75 years.

Etiologies of ESRD and co-morbidities were demonstrated to determine anaemia control in this study. Diabetes (as a primary renal diagnosis and concomitant disease) was found to influence Hb to higher values and EPO delivery to a lower dose. Glomerulonephritis influenced EPO to a higher dose, but not Hb. Theses findings were unexpected. ESAM 2003 showed that patients with diabetes were less likely to achieve Hb ≥ 11 g/dL than those with other primary renal diagnosis and concomitant diseases. The reason why diabetic patients have a higher Hb in our study is not fully explained. However, in our study, patients with diabetes had a significantly higher body weight than non diabetic patients (78.0 ± 15.3 vs 68.0 ± 9.4 kg; p < 0.0001). A higher fat mass may stimulate erythropoiesis through increased endogenous erythropoietin synthesis and/or sensitivity via the adipocyte hormone leptin [15,16]. We indeed observed a significant positive correlation between body weight and Hb (p: 0.0255, r = 0.115). It is thus speculated that the higher fat mass in our diabetic patients might be responsible for their increased Hb.

Patients with Hb>11 g/dL had a significantly better survival compared to patients with Hb<11 g/dL. A trend towards improved survival was also observed with an Hb cut-off of 12 g/dL. We also assessed the influence of EPO dose on mortality and found a trend for a lower mortality in patients with Hb>11 g/dL and EPO dose <200 IU/kg/week, compared to the same Hb level but EPO dose ≥ 200 IU/kg/week. These results are consistent with those in DOPPS II, showing a mortality risk to be 5% lower for every increase of 1 g/dL in Hb concentration. Our data are also in line with the large analysis of Ofsthun et al [17], which showed a mortality benefit for patients beyond the current recommended Hb target. However, we are aware that observational data, such as those presented in this study, is not sufficient to determine the optimal Hb target. Indeed, prospective randomized controlled trials showed that complete normalization of Hb from 126 to 135 g/dL has adverse effects on mortality and morbidity parameters such as blood pressure control in chronic kidney disease stages 3 and 4 [18,19], and on arterio-venous access thrombosis and mortality in dialysis patients [20]. In contrast, quality of life is improved when Hb is normalised [18]. Thus, a long-standing controversy still exists, especially with regard to the upper limit of Hb concentration to suggest [21]. The Hb target aimed by Swiss nephrologists in the current study ranged from 11 to 13,5 g/dL, probably reflecting this controversy.

This survey has several limitations: First, although representing 14% of the Swiss dialysis population, it includes a relatively small number of patients, which limits its statistical power. Second, we did not look at several parameters known to influence Hb level and EPO dose, such as residual renal function, adequacy parameters, inflammation/infection, nutritional parameters, treatment with ACE inhibitors, angiotensin II receptor antagonists and level of iPTH. Third, this is an observational study and even though we paid special attention in order to avoid selection bias, this can't be formally excluded, as there was no random selection into the study. Although exclusion criteria were respected, some unstable patients may have been excluded. This might theoretically have affected our data in a positive fashion. In contrast, some other factors may have had a negative impact on the results: No minimal time on dialysis was requested. It is known that new incident dialysis patients have a lower Hb. We have included twenty EPO-naive patients, who, in general, had a lower Hb than EPO pre-treated patients. Whether the inclusion of these patients had an influence on mean Hb was not tested. Nevertheless, in spite of the above limitations, patients included in this study have similar characteristics/co-morbidities compared to ESAM 2003 [7]. With respect to the exclusion criteria, the only selection made was the exclusive use of epoetin beta, as stated in the inclusion criteria. Therefore, the hypothesis that a selection bias may have significantly affected our results in a positive fashion appears unlikely.

## Conclusion

In comparison to ESAM 2003 and DOPPS II, this survey shows an optimal and sustained quality control of anaemia in a cohort of dialysis patients in Switzerland, followed prospectively for 12 months. Such a control was reached through doses of EPO beta in the upper range of EBPG recommendations, preferential use of subcutaneous administration of EPO and careful management of iron therapy.

## Competing interests

Data collection in the dialysis centres was supported by Roche Pharma Switzerland.

## Authors' contributions

CMM and DT have contributed equally to this article, they analysed and interpreted the data and wrote the article. NL and MB conceived, designed and conducted this survey and corrected the manuscript. LG, DK, PYM were involved in collecting and interpreting data. DG helped to draft the manuscript.

## Pre-publication history

The pre-publication history for this paper can be accessed here:


